# Insights into the migration of the European Roller from ring recoveries

**DOI:** 10.1007/s10336-016-1374-y

**Published:** 2016-08-10

**Authors:** Tom Finch, Jamie Dunning, Orsolya Kiss, Edmunds Račinskis, Timothée Schwartz, Laimonas Sniauksta, Otto Szekeres, Béla Tokody, Aldina Franco, Simon J. Butler

**Affiliations:** 1 School of Biological Sciences, University of East Anglia, Norwich, NR4 7TJ UK; 2 School of Environment and Life Sciences, University of Salford, Peel Building, Salford, M5 4WT UK; 3 Faculty of Agriculture, University of Szeged, Andrássy Street 15, Hodmezovasarhely, H-6800 Hungary; 4 Latvian Ornithological Society, A.k. 105, Riga, LV-1046 Latvia; 5 A Rocha France, 233 Route de Coste Basse, 13200 Arles, France; 6 Naugarduko St. 47-3, LT-03208 Vilnius, Lithuania; 7 “Riparia” Association of Naturalists, Radanovac 83/b, 24000 Subotica, Serbia; 8 BirdLife Hungary, Költo utca 21, Budapest, 1121 Hungary; 9 School of Environmental Sciences, University of East Anglia, Norwich, NR4 7TJ UK; 10 Present Address: RSPB/UCCRI, The David Attenborough Building, Pembroke Street, Cambridge, CB2 3QZ UK

**Keywords:** *Coracias garrulus*, Juvenile, Mortality, Tracking

## Abstract

Despite recent advances in avian tracking technology, archival devices still present several limitations. Traditional ring recoveries provide a complementary method for studying migratory movements, particularly for cohorts of birds with a low return rate to the breeding site. Here we provide the first international analysis of ring recovery data in the European Roller *Coracias garrulus*, a long-distance migrant of conservation concern. Our data comprise 58 records of Rollers ringed during the breeding season and recovered during the non-breeding season. Most records come from Eastern Europe, half are of juveniles and over three quarters are of dead birds. Thus, ring recoveries provide migration data for cohorts of Rollers—juveniles and unsuccessful migrants—for which no information currently exists, complementing recent tracking studies. Qualitatively, our results are consistent with direct tracking studies, illustrating a broad-front migration across the Mediterranean Basin in autumn and the use of the Arabian Peninsula by Rollers from eastern populations in spring. Autumn movements were, on average, in a more southerly direction for juveniles than adults, which were more easterly. Juvenile autumn recovery direction also appeared to be more variable than in adults, though this difference was not statistically significant. This is consistent with juveniles following a naïve vector-based orientation program, and perhaps explains the ‘moderate’ migratory connectivity previously described for the Roller. In the first (qualitative) analysis of Roller non-breeding season mortality, we highlight the high prevalence of shooting. The recovery age ratio was juvenile-biased in autumn but adult-biased in spring. Although not statistically significant, this difference points towards a higher non-breeding season mortality of juveniles than adults. Our study demonstrates the complementarity of ring recoveries to direct tracking, providing an insight into the migration of juvenile Rollers and non-breeding season mortality.

## Abstract (de)

**Durch Ringfunde gewonnene Einblicke in den Zug der Blauracke**

Trotz der jüngsten Fortschritte in der zum Verfolgen der Bewegungen von Vögeln verwendeten Tracking-Technologie sind archivalischen Geräten nach wie vor gewisse Grenzen gesetzt. Traditionelle Ringfunde stellen eine ergänzende Methode zur Untersuchung von Zugbewegungen dar, insbesondere für Kohorten von Vögeln mit geringen Rückkehrraten ins Brutgebiet. Hier stellen wir die erste internationale Analyse von Ringwiederfunden bei der Blauracke *Coracias garrulus*, einem Langstreckenzieher, der für den Artenschutz von Interesse ist, vor. Unsere Daten umfassen 58 Belege von während der Brutsaison beringten und außerhalb der Brutsaison wiedergefundenen Blauracken. Die meisten Belege stammen aus Osteuropa, zur Hälfte von Jungvögeln und zu über drei Vierteln von Totfunden. Daher liefern Ringfunde Zugdaten für solche Blaurackenkohorten, für die derzeitig keine Information vorliegen (Jungvögel und erfolglose Zugvögel), und ergänzen so direkte Trackingstudien. Qualitativ stimmen unsere Ergebnisse mit direkten Trackingstudien überein und zeigen einen Breitfrontzug über den Mittelmeerraum im Herbst und die Nutzung der Arabischen Halbinsel durch Blauracken aus östlichen Populationen im Frühjahr. Herbstliche Zugbewegungen verliefen im Durchschnitt für Jungvögel in südlicherer Richtung als für Altvögel, deren Bewegungen stärker östlich verliefen. Die Richtung der Wiederfunde im Herbst schien für Jungvögel außerdem variabler zu sein als für Altvögel, obwohl dieser Unterschied nicht statistisch signifikant war. Dies steht im Einklang damit, dass die Jungvögel einem naiven vektorbasierten Orientierungsprogramm folgen, und erklärt vielleicht die mäßige Zugkonnektivität, die bislang für die Blauracke beschrieben worden ist. In einer ersten (qualitativen) Analyse der Blauracken-Mortalität außerhalb der Brutsaison betonen wir das starke Überhandnehmen von Abschüssen. Das Altersverhältnis der Wiederfunde war im Herbst zu Jungvögeln und im Frühjahr zu Altvögeln hin verschoben. Obwohl dieser Unterschied nicht statistisch signifikant war, deutet er auf eine höhere Mortalität außerhalb der Brutsaison für Jungvögel verglichen mit Altvögeln hin. Unsere Studie zeigt, wie Ringfunde direkte Trackingstudien ergänzen können, und liefert Einblicke in den Zug junger Blauracken sowie in die Mortalität außerhalb der Brutsaison.

## Introduction

The miniaturisation of animal tracking technology is revolutionising our understanding of avian migration. Lightweight solar geolocators (Ouwehand et al. 2015) and global positioning system tags (Hallworth and Marra 2015) now allow researchers to track the migration of all but the smallest of songbirds. However, these technologies are not without limitations (Blackburn et al. 2016). Most notably, archival loggers must be recovered for data retrieval. As a result, information on the movements of juveniles (which generally disperse further than adults, so are rarely targeted for archival tagging) and unsuccessful migrants (which, by definition, do not return to the tagging site) are rare. Most data on juvenile migration and migration-related mortality are therefore restricted to large-bodied taxa capable of bearing satellite transmitters (but see e.g. McKinnon et al. 2014). Traditional mark-recapture techniques therefore provide a complementary method for studying migratory movements (Reichlin et al. 2008; Panuccio et al. 2013). In particular, ring recoveries provide a good opportunity to study juvenile movements (Thorup et al. 2003a) and causes and rates of mortality (McCulloch et al. 1992).

Describing the spatio-temporal distribution of migrant bird populations at all ages and throughout their annual cycle is particularly pertinent given their widespread decline (Sanderson et al. 2006; Vickery et al. 2013). In particular, understanding the processes by which naïve first-year migrants navigate to and from their first winter site [to which they will usually return with high fidelity in subsequent years (e.g. Blackburn and Cresswell 2016)] is crucial for understanding patterns of connectivity and predicting the response of migratory populations to environmental change (Cresswell 2014). Additionally, knowing where and under what circumstances migrants die contributes to our understanding of how population size is regulated throughout the annual cycle (Strandberg et al. 2009; Klaassen et al. 2014).

The European Roller (*Coracias garrulus*, hereafter ‘Roller’) is a long-distance migrant bird of conservation concern across much of its range (Birdlife International 2015). Although most authors attribute this species’ decline to agricultural change in its breeding range (e.g. Avilés and Parejo 2004), threats on migration and over winter have also been suggested (Kovacs et al. 2008), and the Roller is listed in Appendix II of the Convention on Migratory Species. Until recently, non-breeding-season threats were difficult to assess due to our limited understanding of Roller migration. However, adult Rollers from across their European range have now been tracked to and from their Southern African winter sites using solar geolocators (Emmenegger et al. 2014; Catry et al. 2014) and platform transmitter terminal satellite tags (Rodríguez-Ruiz et al. 2015), revealing the degree of connectivity between breeding and wintering sites (Finch et al. 2015).

Here, we complement these tracking studies with a coordinated international analysis of ring recoveries [as advocated by e.g. Bairlein (2001)], the first of its kind for this species. Specifically, we compare age-related differences in autumn-recovery direction, and seasonal differences in recovery age ratio. We expect juvenile autumn migration to be in a variable but, on average, southerly direction (e.g. Perdeck 1958), and the ratio of juvenile to adult recoveries to decrease between autumn and spring migration (e.g. Johnson 1973). We also describe causes of mortality during the non-breeding season.

## Methods

In order to study the migratory movements of the Roller we collated all known records of Rollers ringed in Europe and recovered, recaptured or resighted away from their original capture site. Records acquired from the EURING Data Bank (du Feu et al. 2009, extracted 16 December 2015) were supplemented with additional ring recoveries from national schemes in Hungary, Latvia, France, Bulgaria, Serbia and Lithuania.

We restricted our dataset to birds ringed during the breeding season (June–August) and assume that ringing sites represent natal/breeding origin and that recovery sites represent a single point along the (successful or otherwise) migration route. In the case of birds recovered after the year of ringing, we assume that Rollers are philopatric to their original ringing (i.e. hatching/breeding) site. The exclusion of birds ringed as juveniles and recaptured as adults (i.e. where the breeding site is uncertain due to natal dispersal) was not possible, as these made up 88 % of adult recoveries. Although quantitative data on Roller natal philopatry are limited, we have numerous anecdotal records from populations across Europe of ringed Rollers breeding <1 km from their natal site, and only a handful of records of Rollers dispersing to breed further afield [the record is 334 km from France to Hungary (Vincent-Martin et al. 2013)]. As in other species (Paradis et al. 1998), breeding dispersal is believed to be substantially lower than natal dispersal, with Rollers often nesting in the same cavity in subsequent years. The potential for natal dispersal to affect our results (by causing us to misidentify true breeding sites) is unavoidable.

Following Reichlin et al. (2008), we limited recoveries to those exceeding 100 km from the original ringing location in an attempt to exclude short-distance pre-migratory movements. In accordance with Cramp (1985) and Finch et al. (2015), we assigned recoveries to one of three seasons; autumn migration (August–November, inclusive), winter (December–February) and spring migration (March–May). Recoveries in June and July were excluded, as these are unlikely to represent migratory movements. Birds recovered during their first autumn or spring migration were classed as ‘juvenile’ and otherwise as ‘adult’. EURING data were read into R (R Development Core Team 2014) using the birdring package (Korner-Nievergelt and Robinson 2015), and recovery direction was calculated using the geosphere package (Hijmans 2015). Condition (dead, alive or sick) and circumstances [shot, collision (traffic or other), resighted or recaptured] were acquired when known.

We compared the relative frequency of juveniles and adults (i.e. recovery age ratio) in autumn and spring using a *Χ* ^2^ contingency test. Autumn recovery direction was clustered towards the south (mean = 177°) and approximately normally distributed, so was treated as a linear variable rather than circular one. We compared adult and juvenile autumn recovery angle using a Welch’s *t*-test, and variance in autumn recovery angle using an ANOVA.

## Results

A total of 58 recoveries met our specifications: 11 from the EURING Data Bank, and 47 from national schemes in Hungary (*n* = 18), Latvia (16), France (5), Bulgaria (3), Serbia (3) and Lithuania (2). Of 149 initial records from EURING, we excluded 138; fourteen birds were ringed outside the breeding season, a further 91 were recovered during the breeding season, and of the remaining 44, thirty-three were recovered within 100 km of the ringing site.

The distribution of recoveries over time was distinctly bimodal, with 18 recoveries each from the 1930s and 2010s but only 22 records (mean = 3.1 records per decade) from all intervening decades. Almost all records were from easterly populations, with only six recoveries of birds ringed west of 15°E. The majority of recoveries (41) occurred during autumn migration, with fewer spring recoveries (16) and only one from the sub-Saharan winter area (Table 1).

**Table 1:** Numbers of ringed European Rollers recovered by age (columns) and season (rows)

	Adult	Juvenile	Total
Autumn	15	26	41
Winter	0	1	1
Spring	9	7	16
Total	24	34	58

**Fig. 1:**
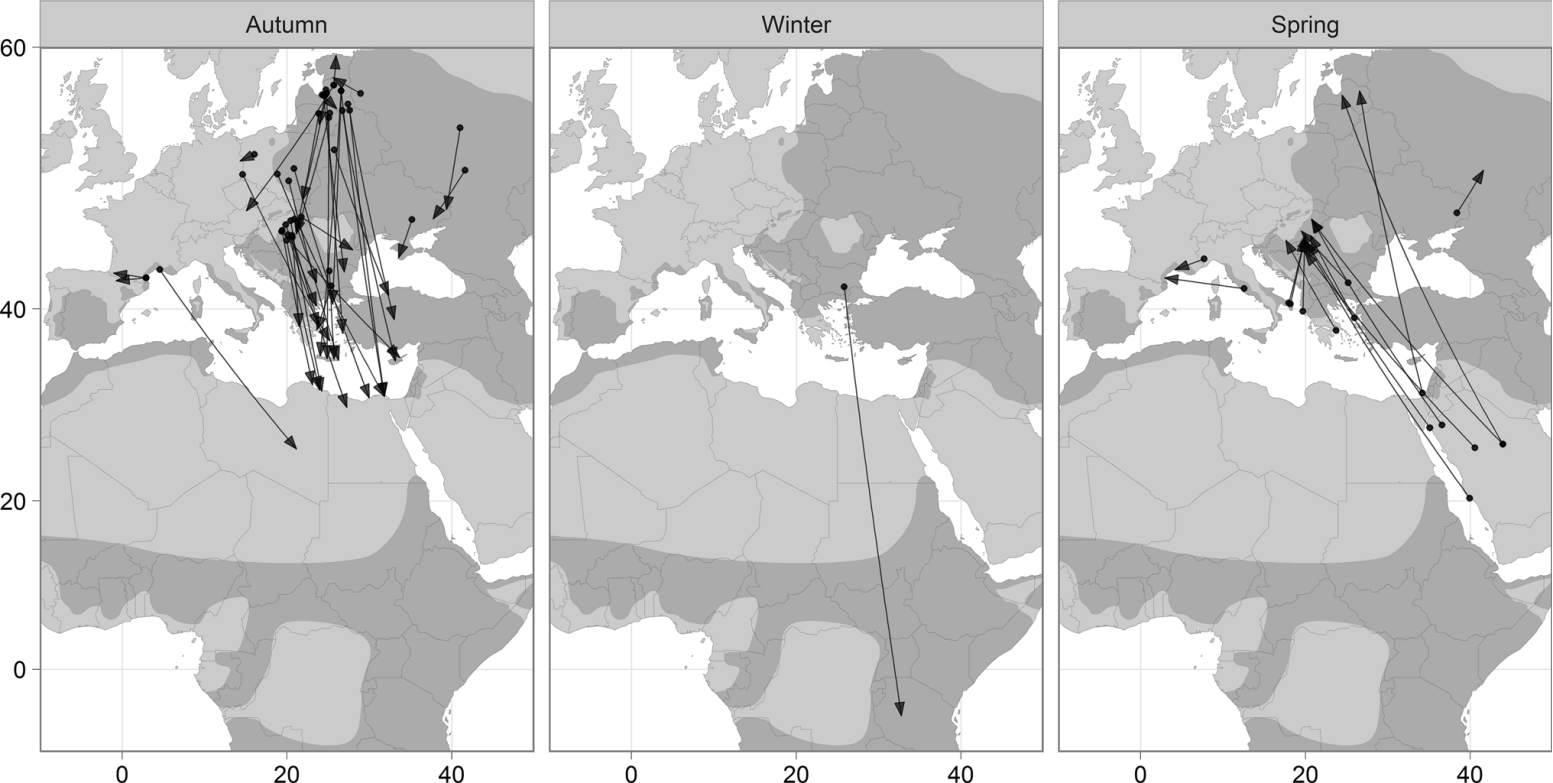
Autumn (*left*), winter (*middle*) and spring (*right*) recoveries of ringed European Rollers. *Arrows* denote direction of movement (from ringing to recovery site in autumn and winter, and recovery to ringing site in spring). *Shaded regions* show the Roller’s distribution during breeding (Europe) and winter (sub-Saharan Africa) seasons (Birdlife International 2013). Mercator projection

Our data illustrate a broad-front southerly passage through the Balkan states and Eastern Mediterranean Basin in autumn (mean ± SE direction from ringing to recovery site = 177° ± 7.3), with spring movements generally originating further east (mean direction from recovery to ringing site = 329° ± 9.1), including several records from the Arabian Peninsula (Fig. 1).

**Fig. 2:**
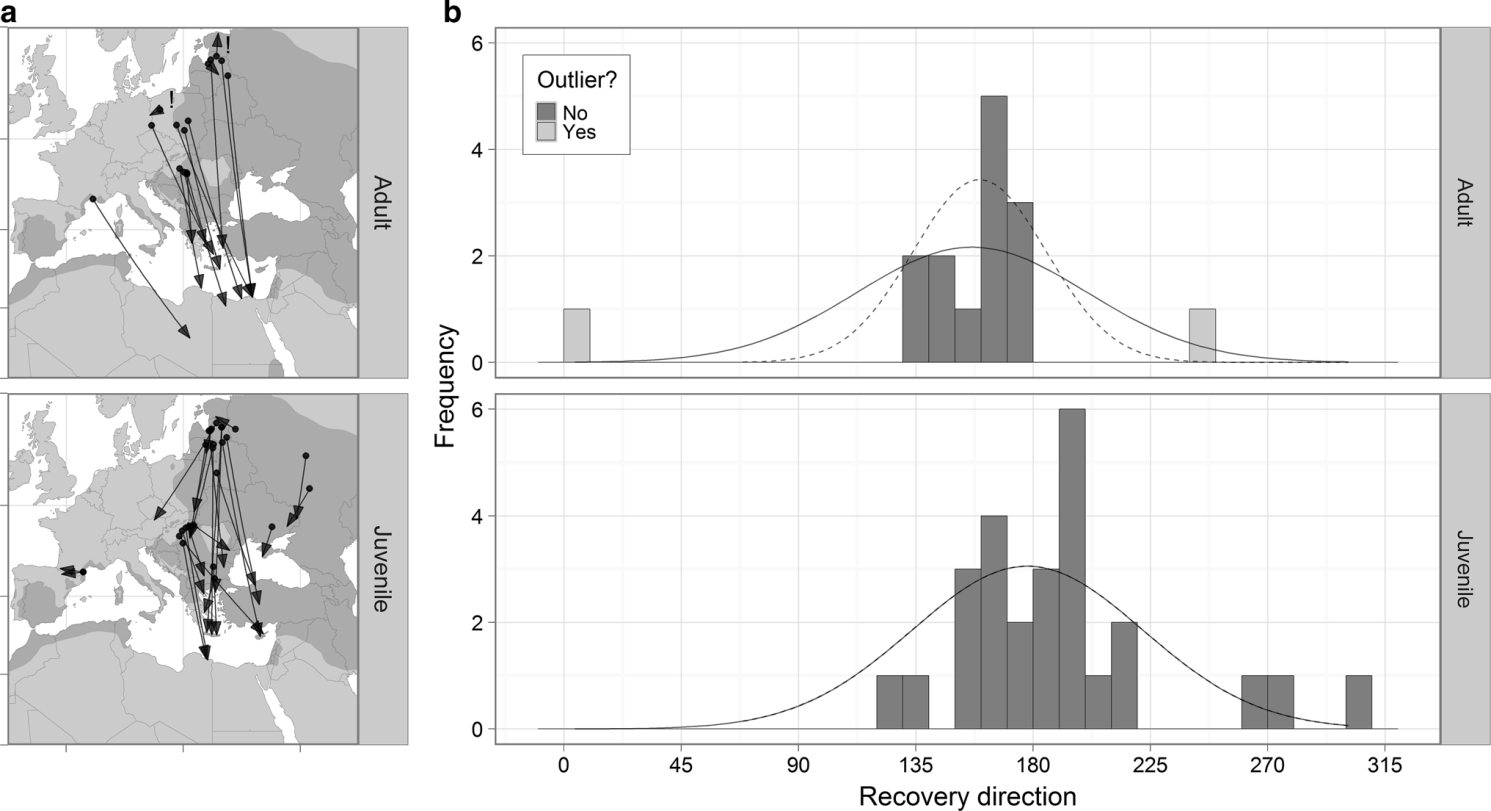
Comparison of adult (*top*) and juvenile (*bottom*) autumn migration. **a** *Arrows* denote direction of movement from ringing to recovery site. **b** Frequency distribution of autumn recovery direction for adults and juveniles. *Curved lines* represent the normal density curve with corresponding mean and SD. Outliers were detected using Rosner’s generalized extreme Studentized deviate test (*k* = 2), and are identified by an *exclamation mark* in **a** and *light shaded bars* and *dashed lines* in **b**

Juveniles made up 63 % of autumn recoveries but only 44 % of spring recoveries (Table 1), though the frequency of recoveries by season and age class did not differ significantly from random (*Χ* ^2^ = 1.1, *df* = 1, *p* = 0.29). Mean autumn recovery direction was more easterly in adults (mean = 154° ± 12.6) than juveniles, which migrated approximately due south (190° ± 8.0; *t* = 2.4, *df* = 25.4, *p* = 0.03; Fig. 2a). There was no difference in the variance of autumn recovery direction between adults and juveniles (*F*-test; *F* = 0.7, *df* = 14, 25, *p* = 0.43; Fig. 2b).

**Fig. 3:**
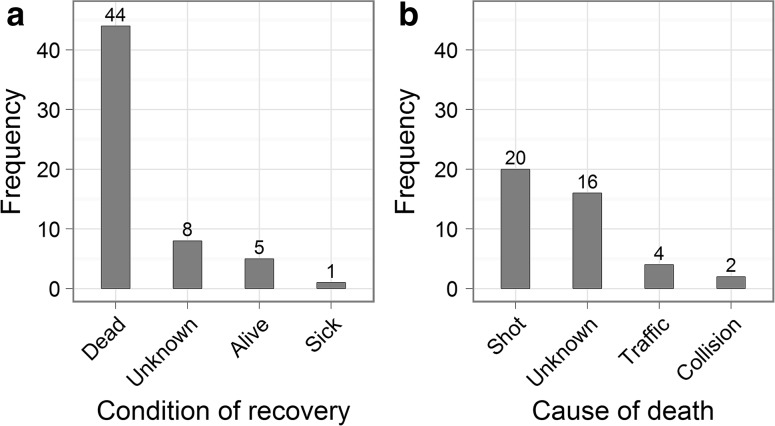
Frequency of Roller recovery records by **a** condition (dead, unknown, alive or sick) and **b** circumstances of death (shot, unknown, traffic or other collisions). Two Rollers ‘recaptured’ as dead birds were excluded from **b**

Most recoveries (76 %) were of dead birds. Shooting was the most common cause of mortality (48 %), followed by traffic casualties (10 %) and other collisions (5 %); the circumstances of death were unknown for 36 % of dead birds (Fig. 3).

## Discussion

We present the first Europe-wide analysis of Roller ring recovery data, complementing several recent tracking studies (Emmenegger et al. 2014; Catry et al. 2014; Finch et al. 2015; Rodríguez-Ruiz et al. 2015). We gathered 57 records of Rollers recovered on migration and one winter record. More than half of the recoveries in our data set are of juvenile migrants during their first autumn or spring migration, and over three quarters are records from dead birds. Neither juveniles nor dead migrants are represented in previous tracking studies, demonstrating the complementarity of ring recoveries to e.g. solar geolocators. We had insufficient data to compare changes in migration over time, but the recent increase in the number of recoveries (two in the 1990s, eight in the 2000s and 18 already in the 2010s) is encouraging.

We did not have access to data on Europe-wide ringing effort, so could not calculate the overall recovery rate. Instead, where available, we can get an indication of recovery rates from national statistics. In France, for example, 1942 Rollers were ringed between 2002 and 2013, five (0.26 %) of which were recovered in circumstances which met our selection criteria. Equivalent recovery rates for Lithuania (1929–2015) and Serbia (2003–2015) were 0.63 and 0.19 %, respectively. Whilst these recovery rates are higher than those reported by Robinson et al. (2009) for a suite of British bird species, we are nevertheless left with a rather small number of recoveries, most of which come from Eastern Europe. This spatial imbalance contrasts with (and therefore complements) recent direct tracking studies, in which most data came from western populations (Finch et al. 2015). Whilst the western bias of tracking studies is probably a general pattern reflecting funding inequalities, the eastern bias of ring recoveries is likely specific to the Roller. The Roller’s distribution in Western Europe is both restricted and southerly (Fig. 1), such that the passage of western Rollers through Europe (where recovery rates are relatively high) is limited. Thus, our conclusions are principally limited to the migration of Rollers from Eastern Europe.

Without quantitative data on spatio-temporal variation in recovery probability, we refrain from making a formal comparison with previous direct tracking data. However, our results are qualitatively consistent with Finch et al. (2015), with autumn recoveries from the Balkan Peninsula, Libya and Egypt illustrating a broad-front migration of Rollers from central and eastern populations across the Mediterranean Basin. We also demonstrate that individuals from Hungary and Serbia—in addition to Latvia, as revealed here and by solar geolocators (Finch et al. 2015)—migrate through Arabia in spring. Anecdotal evidence suggests that a large number of Rollers are shot in Arabia on spring migration (del Hoyo et al. 2001), as were four out of the six ringed Rollers recovered in this region.

Only one ringed Roller has been recovered in its sub-Saharan winter quarters. Evidently, ring recoveries are not an effective way of describing the Roller’s winter distribution, presumably due to low encounter and/or reporting rates of ringed birds in sub-Saharan Africa (Clark et al. 2009; Thorup and Conn 2009).

### Age differences

Ring recoveries provide the first chance to study the migratory movements of juvenile Rollers which, due to their low return rate to the natal site, have yet to be tracked with archival solar geolocators (or otherwise). Autumn recovery direction was significantly higher (i.e. more southerly) for juveniles than adults, though variance in autumn recovery direction did not differ significantly between age classes. Nevertheless, with the exception of two adult recoveries, all remaining autumn recoveries fell in a narrow directional band for adult birds (between 133° and 175°), whereas juvenile recoveries were more inconsistent (between 120° and 280°; Fig. 2). These two adults—ringed as chicks and recovered 4–9 years after ringing and <220 km away—migrated in a northerly and west-south-westerly direction, respectively. We have assumed that the origin of these migratory movements is the initial ringing site, which in most cases was the natal site (because most birds were ringed as nestlings). However, given that natal dispersal is unmeasured, we cannot be certain that individuals ringed as chicks but recovered as adults are not migrating to/from a site removed from their place of ringing. This uncertainty is likely to influence most the direction of short-distance recoveries, potentially justifying the exclusion of these two individuals [in the bottom 15 % of adult autumn recovery distance, and recovered less than the maximum recorded natal dispersal distance from their natal site (Vincent-Martin et al. 2013)].

These results—juveniles orientating variably but, on average, due south—are consistent with the current state of knowledge on avian navigation and orientation, though we urge caution given our limited sample size. Displacement experiments suggest that whilst adult migrants are goal-oriented (using ‘map’ information acquired on previous journeys), juveniles migrate using simple compass-based vector navigation and are unable to compensate for artificial displacement (Perdeck 1958; Thorup et al. 2007). Orientation studies have found that juvenile orientation is less precise (Holland and Helm 2013) and high-resolution tracking shows that juveniles are more susceptible to wind drift (Thorup et al. 2003b). As a result, the migration routes of juveniles tend to be more tortuous than those of adults (e.g. Mellone et al. 2013), and their ultimate selection of winter sites may be more stochastic (reviewed by Cresswell 2014). Assuming that successful juveniles return to their first winter site as adults, this pattern of variable juvenile orientation could explain the ‘moderate’ connectivity observed by Finch et al. (2015), in which individual Rollers from different breeding populations do not occupy distinct non-breeding quarters, instead overlapping with individuals from other (often distant) breeding populations.

In autumn, juvenile recoveries were 1.73 times more frequent than adult recoveries, presumably due to the greater abundance of young birds immediately following the breeding season (and the fact that nestlings are probably more frequently ringed than breeders). In spring, however, the age ratio was adult-biased (0.78). We tentatively argue that the lower relative recovery rate of juveniles in spring suggests a lower non-breeding survival in juveniles compared to adults, though these differences were not statistically significant. More formal studies of mortality during migration, though rare, generally show higher mortality in juveniles compared to adults (Johnson 1973; Owen and Black 1989; Strandberg et al. 2009; Guillemain et al. 2010; but see Grüebler et al. 2014).

### Mortality

In contrast to archival solar geolocators, which record only successful migrations, 76 % of ring recoveries were of dead birds, presenting a rare opportunity to explore the causes of mortality during the migration of Rollers. Cause of death was unknown in 36 % of cases, but 48 % of birds were shot. Due to limited sample size it is difficult to quantify spatial and temporal variation in hunting pressure, though all shooting records came from Eastern Europe, North Africa or Saudi Arabia.

As with all conclusions based on ring recoveries, it is important to bear in mind potential recovery biases when assessing causes of mortality. Birds dying of natural causes are less likely to be encountered and reported, so anthropogenic causes of death are probably over-represented in our database (Clark et al. 2009). Nevertheless, (illegal) hunting is likely to have a lower reporting rate than other anthropogenic causes of mortality, so we highlight the high prevalence of shooting in our dataset as being of real concern. A recent analysis of illegal hunting in the Mediterranean highlighted the European Roller as one of 20 species of conservation concern with the highest estimated number of birds killed (relative to population size), with the greatest numbers taken in Syria, Cyprus and Lebanon (Brochet et al. 2016). Hunting impact has not been estimated for Arabia, but in North Sinai (Egypt) it is estimated that over 400 Rollers are trapped annually in trammel nets, a cause of mortality not represented in our dataset (Eason et al. 2016).

### Conclusion

By collating ring recovery data from across the Roller’s European range, we present the first glimpse into the autumn migration of first-year Rollers. Movements of juveniles were more southerly and, after the exclusion of (adult) outliers, more variable than those of adults, consistent with juveniles following a naïve vector-based orientation program. We also provide the first study of causes and rates of non-breeding season mortality, highlighting the prevalence of shooting as being of particular concern.
